# Functional aspects of primary cilia in signaling, cell cycle and tumorigenesis

**DOI:** 10.1186/2046-2530-2-6

**Published:** 2013-04-29

**Authors:** Sander G Basten, Rachel H Giles

**Affiliations:** 1 Department of Medical Oncology, UMC Utrecht, Universiteitsweg 100, Utrecht, 3584 CG, The Netherlands; 2 Department of Nephrology and Hypertension, University Medical Center Utrecht, Heidelberglaan 100, Utrecht, F03.223, 3584 CX, The Netherlands

**Keywords:** Cilia, Signal transduction, Cell cycle, Cancer

## Abstract

Dysfunctional cilia underlie a broad range of cellular and tissue phenotypes and can eventually result in the development of ciliopathies: pathologically diverse diseases that range from clinically mild to highly complex and severe multi-organ failure syndromes incompatible with neonatal life. Given that virtually all cells of the human body have the capacity to generate cilia, it is likely that clinical manifestations attributed to ciliary dysfunction will increase in the years to come. Disputed but nevertheless enigmatic is the notion that at least a subset of tumor phenotypes fit within the ciliopathy disease spectrum and that cilia loss may be required for tumor progression. Contending for the centrosome renders ciliation and cell division mutually exclusive; a regulated tipping of balance promotes either process. The mechanisms involved, however, are complex. If the hypothesis that tumorigenesis results from dysfunctional cilia is true, then why do the classic ciliopathies only show limited hyperplasia at best? Although disassembly of the cilium is a prerequisite for cell proliferation, it does not intrinsically drive tumorigenesis *per se*. Alternatively, we will explore the emerging evidence suggesting that some tumors depend on ciliary signaling. After reviewing the structure, genesis and signaling of cilia, the various ciliopathy syndromes and their genetics, we discuss the current debate of tumorigenesis as a ciliopathy spectrum defect, and describe recent advances in this fascinating field.

## Review

### Ciliogenesis

**Figure 1:**
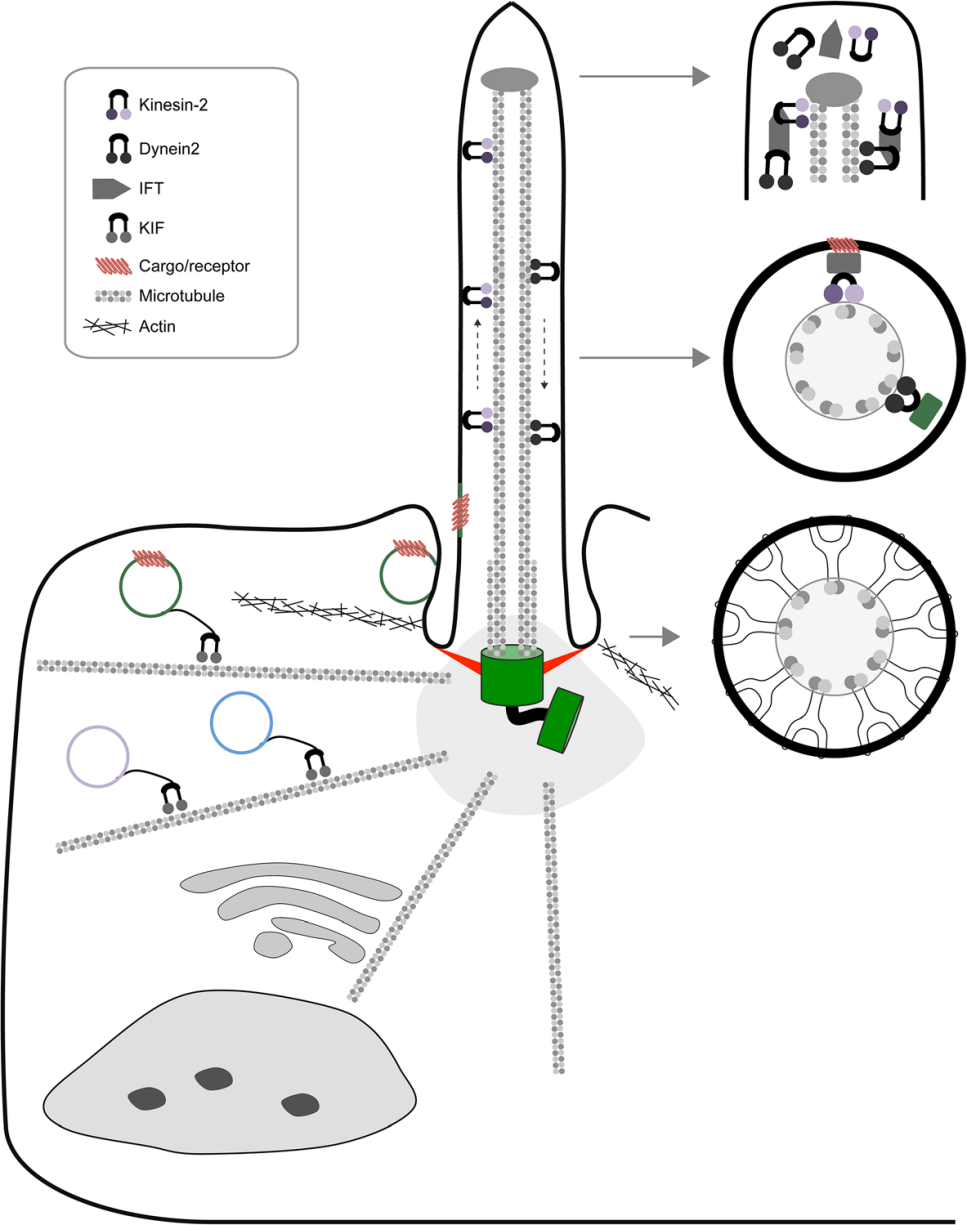
**Structure of the primary cilium. **The two centrioles are surrounded by the pericentrosomal matrix, serving as basal body and microtubule organizing center. Vesicular transport delivers ciliary components either to the basal body or ciliary pocket. Axonemal import is regulated by the transition zone, which is marked by Y-links. Anterograde kinesin-2- and retrograde dynein-2-mediated IFT sustain ciliary maintenance and cilia-dependent signaling; the complex rearranges at the ciliary tip.

Once a cell enters quiescence, it must go through a series of events to realize a fully matured cilium. All cilia subtypes must conform to a basic structure. The elemental cilium is composed of the basal body (BB) that is located at the apical plasma membrane, and an axoneme that forms a thin projection extending from the membrane (Figure 1) [1]. The BB is derived from the centrosome and comprises a mature centriole, accessorized by sub-distal appendages, connected to an immature centriole that is surrounded by a dense protein-rich pericentriolar matrix [2]. In early ciliogenesis, a golgi-derived ciliary vesicle fuses with the BB at the distal side of the mature centriole that features the appendages and CEP164 seems to act as the principal linker between the centriole and ciliary vesicle [3]. After the basal body has migrated to the plasma membrane, a predominantly actin cytoskeleton-mediated process [4], the additional structures allow proper docking at the cell membrane, a complex process that requires the interplay of at least Ofd1, Ofd2, Ninein, Mks1, Mks3, Cep164, Poc5 and Cep123, and is extensively reviewed by Reiter, Blacque and Leroux [5]. Initial invagination and centriolar elongation of the ciliary vesicle occurs prior to membrane docking in most cell systems, but can also occur in later stages. Correct lengthening of the mature centriole is critical, and is ensured by interplay of a network of proteins including CP110, CEP97, KIF24 and TTK2 [6,7]. Once the BB has docked and fused with the plasma membrane, growth of the axoneme can be initiated, a process fueled by targeting additional vesicles to the BB. Interestingly, the efficacy of this growth is highly dependent on the dynamics of local actin filaments [8].

Polarized vesicle transport to the BB is an extremely complex process and only a few of the proteins/events required have yet been identified. Firstly, microtubules must extend from microtubule organizing center and link the golgi-ER to the basal body, the microtubule- stabilizing and nucleating proteins EB1 and EB3 are required for microtubule minus-end anchoring, defects in which result in defective cilia biogenesis [9]. BB-targeted vesicles are covered with coat-proteins for specificity including members of the TRAPII, clathrin and exocyst systems (reviewed by Hsiao, Tuz and Ferland) [10]. Perhaps the best-studied example of vesicle transport is the BBSome; a protein complex that forms a cilia-specific transport module [11]. An additional regulatory layer is mediated by Ras-superfamily GTPases of Rab and Arl/Raf subtypes. These molecular switches can undergo conformational change upon binding or hydrolyzing GTP, a process regulated by guanine exchange factors (GEF) and guanine activating proteins (GAP). For example, Rab8, Rab11 and the GEF Rabin8 regulate transport of the BBSome [12]. Alternate cilia-associated vesicle targeting GTPases are Arl3, Arl6 (BBS3) and Arl13 [10]. Finally, kinesin molecular motors transport the vesicles. Beyond the well-described kinesin-2 motor complex, little is known about other members of the large and diverse superfamily of kinesins that contribute to ciliogenesis. Importantly, cilia are highly dynamic structures requiring a continuous supply of molecules for their maintenance [13]. Vesicles can alternatively dock to the ciliary pocket, a highly specialized endocytic membrane domain characterized by an invagination of the cell membrane peripheral to the axoneme [14]. The ciliary pocket is thought to be a zone of excessive crosstalk between the ciliary membrane and plasma membrane characterized by the presence of many endocytosis-associated clathrin-coated pits [15]. Finally, vesicles could potentially dock to the ciliary rootlet; a basal body-associated structure that extends into the cytosol which can at least interact with kinesin light chain subunits [16].

The transition zone (TZ) is just distal of the mother centriole and forms a barrier to regulate protein entry into the cilium [17]. Structurally, the TZ is composed of transition fibers, the ciliary necklace and Y-fibers essential for membrane anchoring and formation of a selectively permeable pore (reviewed by Garcia-Gonzales and Rieter [17]). Size-exclusion prevents large structures from entering the axoneme [18]. In addition, components of IFT complexes have been reported to dock to these fibers and possibly participate in regulation of ciliary entry [19]. The Y-links are composed of protein networks, many members of which are encoded for by classic ciliopathy disease loci (described below). Located adjacent to the axonemal microtubules is a core protein complex consisting of NPHP1, NPHP4 and NPHP8 [20], which interacts with a second core complex termed the MKS-JBTS module [21]. The MKS-JBTS module contains distinct proteins, many of which have lipid-binding domains (C2, B9 domains) including MKS1, B9D1 and B9D2; these proteins interact with the ciliary membrane [17]. Anchoring to the membrane requires transmembrane domain-containing proteins (TMEM), such as TMEM216 [22] and TMEM237 [23] and interacting proteins TMEM67 [24], TMEM231 [25] and putatively TMEM107 [26]. Moreover, SEPT2 forms a diffusion barrier at the base of the cilium [27]. An emerging aspect of regulated cilia entry is a proposed import system that is analogous to and overlaps with the nuclear pore complex. This so-called ciliary pore complex contains well-known members of the nuclear pore complex, including Ran-GTPases, importins and nucleoporins which are known to localize to the ciliary base [18]. In line with this notion, some proteins contain ciliary localization sequences, including PC1 [28], fibrocystin [29], KIF17 [30], gpr161 [31] and some myristoylated proteins such as NPHP3 [32].

Nine highly stable axonemal microtubule doublets serve as major transport fibers for intraflagellar transport (IFT), an exclusively ciliary transport mechanism that was identified by pioneering studies of the Rosenbaum lab [33]. Kinesin-2, composed of KIF3A, KAP3 and KIF3B or KIF3C [34], transports selected cargo and dynein-2 during anterograde IFT transport towards the ciliary tip. The complex rearranges at the tip where dynein-2 becomes activated, facilitating retrograde IFT transport [13]. Both kinesin-2 and dynein-2 transport a large protein complex that contains two biochemically and functionally distinct core sub-complexes termed IFT-A and IFT-B. The IFT-B complex is composed of 14 members, including the hallmark IFT88 protein [13]. Dysfunctional IFT-B proteins typically disturb axonemal growth, indicating that this complex mainly functions in anterograde transport. In contrast, the six IFT-A members seem to be mainly involved in retrograde transport; accordingly, defects in IFT144 as well as dynein-2 lead to accumulation of IFT particles at the ciliary tip, generating a bulge [35]. It should be stressed that this view is a simplification of the reality; certain members of both complexes are known to cause inconsistent phenotypes. The core IFT-A, IFT-B, and either kinesin-2 or dynein-2 complexes are thought to facilitate import across the transition zone as well as distribution of cargo-proteins along the axoneme [13]. Currently, we are only beginning to understand the full complexity of achieving specific ciliary import and distribution. Moreover, it is not entirely clear which IFT components are essential or dispensable for cilia formation. Finally, once the primary cilium is matured, constant maintenance is required to render cilia functional. As protein synthesis is absent in the axoneme, ciliary components need to be constantly imported and exported, a function which is highly dependent, but not exclusively restricted to the kinesin-2/IFT system [33]. A number of alternative kinesins appear to have an accessory role in cilia function. This includes KIF17, the ortholog of *Caenorhabditis elegans* OSM-3, a co-factor in axonemal transport required for distal end formation in a subset of sensory cilia [34]. While vertebrate KIF17 appears mostly required for targeting specific ciliary components and for photoreceptor outer segment function, some results are contradictory [36,37]. Another kinesin implicated in cilia function, but not essential for cilia structure or morphology, is the *Caenorhabditis elegans* gene *klp-6*, encoding a kinesin-3 member that transports mechanosensory polycystins in cilia [38]; however, a mammalian ortholog has not yet been described. Finally, KIF7 is the mammalian ortholog of *Drosophila* Costal2, and an important mediator of sonic hedgehog-signaling in mammals [39]. Although its ciliary transport is pivotal for hedgehog-signaling, it is neither required for cilia formation nor stability [39].

### Specialized cilia function

Generally speaking, cilia transduce signals from extracellular stimuli to a cellular response that regulates proliferation, differentiation, transcription, migration, polarity and tissue morphology [40]. The textbook example is the renal primary cilium; a non-motile sensory monocilium extending from the epithelial apical membrane into the fluid-filled lumen, easily accessible to extracellular modulators such as mechanical forces and freely diffusing biological agents. Similar primary cilia can be found on other epithelia in organs containing tubular or acinar structures such as the pancreas [41], and cells of the central nervous system (CNS) [42]. Cilia expressed in endothelial cells of the cardiovascular system protrude far less into the lumen and are implicated in sensing fluid dynamics [43]. Endothelial cilia appear more submerged in the cell and are characterized by the presence of deep ciliary pockets [15], cumulus cells in developing oocyte structures also exhibit similarly deep ciliary pockets [14]. More specialized types of cilia, such as the retinal-connecting cilium and kinocilia together with actin-based stereocilia, can be found in the visual and auditory systems. An intriguing recent addition to the growing spectrum of ciliary subtypes is the immunological synapse formed by T-cells towards antigen-presenting cells which is highly dependent on IFT proteins and therefore considered a functional homolog of the primary cilium [44]. Although most cilia subtypes function through outside-in sensation, some cilia are able to manipulate the extracellular environment, for example, at the node where their swirling motion induces fluid flow that subsequently asymmetrically deposits morphogens to establish body-axis polarity [45]. Here, cilia motion is achieved through the orchestrated regulation of dynein arm complexes [45]. The regulation of body-axis polarity is however more complex and incompletely understood, and depends on the interplay of centrally placed motile cilia and peripheral mechanosensory primary cilia that asymmetrically display an elevated Ca^2+^ response and corresponding changes in downstream gene expression [45].

Sperm motility is similarly achieved through swirling motion, but these cilia (or flagella) can also display a whip-like beating pattern, attributable to an additional central pair of microtubules. Finally, beating cilia can also be found in ependymal cells, fallopian tube epithelia and epididymis epithelia, generating fluid flow, or, in the trachea, stimulating mucus transport [1]. Despite this large diversity, when pan-ciliary processes are disturbed, multi-organ pathologies arise that are collectively termed ‘ciliopathies’ [46].

### Ciliopathies

In their landmark paper, Pazour and colleagues [47] describe the IFT core component *Ift88* to be essential in *Chlamydomonas reinhardtii* and mouse primary cilia formation, thereby kicking off more than a decade of research that has highlighted the importance of cilia function in development and tissue homeostasis. Given the broad expression and function attributed to cilia [48] it is not surprising that defects in this organelle gives rise to a multitude of organ-specific functional defects and pathologies, most of which are prominent in a number of pleiotropic disease-syndromes.

There are many phenotypes that regularly associate with ciliopathies. Common ciliopathy disease syndromes are Bardet-Biedl syndrome (BBS), Jeune asphyxiating thoracic syndrome (JATD), orofaciodigital syndromes (OFD), nephronophthisis (NPHP), Meckel syndrome (MKS), Senior-Løken syndrome (SNLS), Sensenbrenner, Joubert syndrome (JBTS) Alström syndrome (ALSM), Usher syndrome (USH), Leber congenital amaurosis (LCA), short-rib polydactyly syndromes (SRPS) and Ellis van Creveld syndrome (EVC) [46,49]. Most notably affected is the kidney, which features the development of renal cystic expansion that is comparable to lesions caused by autosomal dominant and recessive forms of polycystic kidney disease (ADPKD, ARPKD) [50], coupled with degeneration and increased fibrosis in the case of nephronophthisis [49]. Cyst formation can also often be observed in the pancreas and liver [51]. Other frequently observed disease manifestations are retinal and auditory defects such as retinitis pigmentosa (RP), retinal dystrophy and sensorineural hearing loss [46]. Severe ciliopathies are characterized by abnormal bone development that can be apparent as short ribs and shortening of the long bones, polydactyly and severe craniofacial malformations [52]. Somewhat overlapping with the craniofacial phenotypes are CNS defects, including encephalocoele, hydrocephalus and cerebellar abnormalities such as corpus callosum aging and cerebellar vermis hypoplasia [46,53]. The capacity of the brain to interpret the senses is often affected in ciliopathies, resulting in neurological disorders; cognitive impairment, anosmia, mental retardation, autism, and obesity are apparent in various degrees in many of the ciliopathies [53]. In line with this hypothesis is the recent study testing genes implicated in the neuropsychiatric disorders schizophrenia, bipolar affective disorder, autism spectrum disorder and intellectual disability; the authors identified 20/41 genes to reduce and 3/41 to increase cilia length [54].

A unique category of ciliopathies is associated with motile cilia dysfunction in primary ciliary dyskinesia (PCD) or Kartagener’s syndrome. Here, primary cilia function without apparent defects, but motile cilia are affected. Failure to generate ciliary motion can cause *situs inversus* (only associated with Kartagener’s syndrome), infertility due to immotile spermatozoa and recurrent airway diseases resulting from suboptimal mucus clearance from the trachea and lungs and subsequent extended exposure to pathogens. Clinical symptoms include chronic otitis media, rhinitis, nasal congestion, sinusitis and bronchiectasis [55].

Large-scale genetic, proteomic, and genotype-phenotype studies have generated an ever-growing list of crucial ciliary mediators. Roughly 1,000 proteins comprise the ciliome, calculated from a combination of proteomics and comparative genomics [56]. There is significant pleiotropy in the various ciliopathy syndromes, as well as a gradual increase in severity of disease, indicating that some cilia processes are only slightly modified, whereas others severely impaired. To date, mutations have been recovered at > 50 ciliopathy disease loci, but novel loci are recovered at a high rate. For extensive reviews on the clinical pathologies and genetics associated with ciliopathies we recommend excellent recent reviews by Tobin and Beales [46], Waters and Beales [49], D’Angelo and Franca [51] and Davis and Katsanis [57].

### Ciliary signaling

The cilium protrudes into the extracellular environment, rendering it susceptible to outside-inside-signaling excited by extracellular cues including flow (kinetic) and morphogenic, olfactory and hormonal (chemical) stimuli. The cilium forms a semi-closed system requiring regulated import, retention, and export of components [17] which allows a controlled regulation of signaling molecules passing through the cilium. In recent years, a number of core signaling pathways have been described to depend on intact cilia.

**Figure 2:**
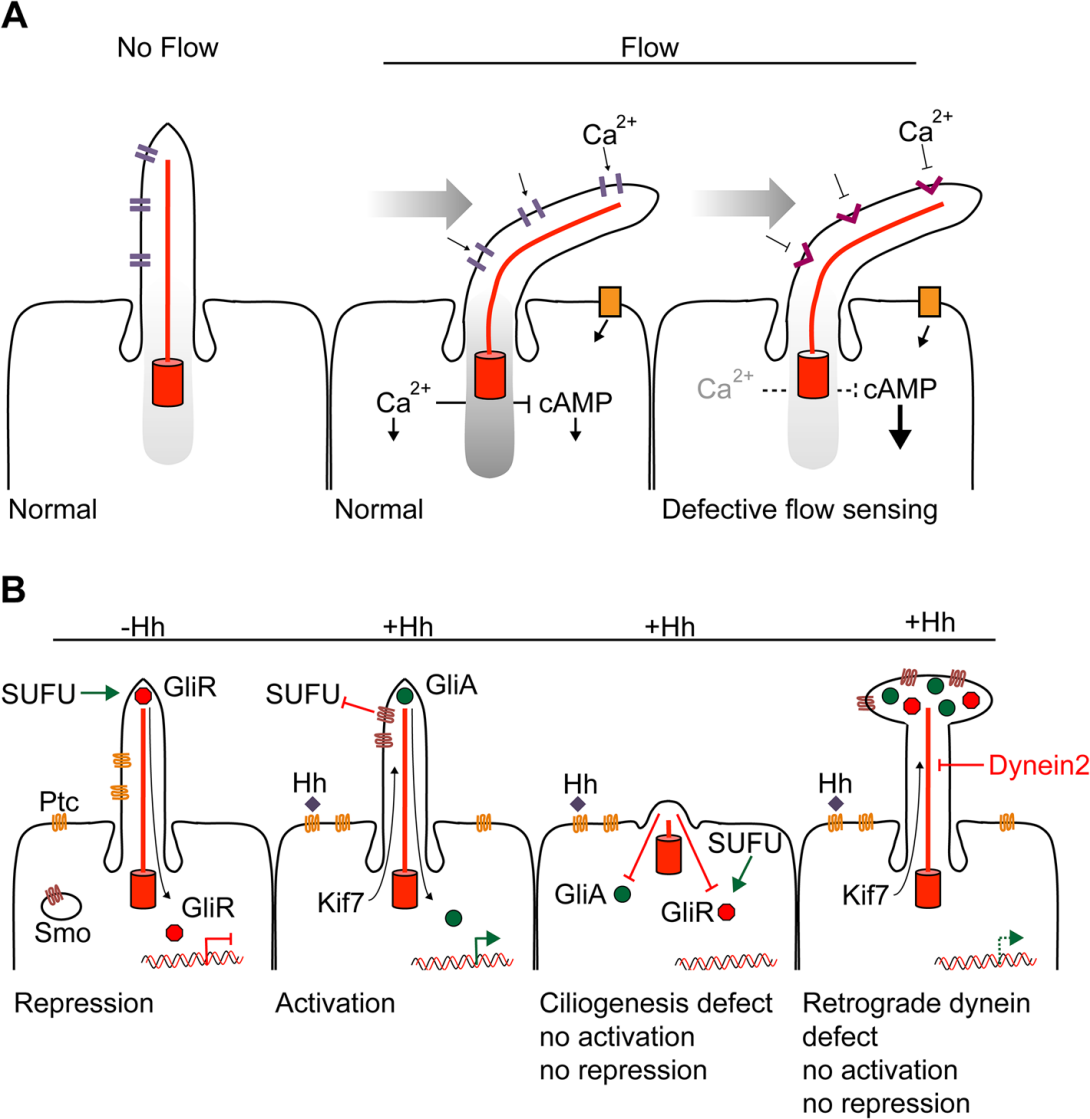
**Ciliary signaling. **(**A**) Mechanosensation. Flow induces cilia bending, the polycystin complex is a Ca^2+ ^channel and causes an increase of intracellular Ca^2+ ^levels that acts as a second messenger. In PKD, the polycystin complex fails to elevate intracellular Ca^2+ ^hence mechanosensation is perturbed, leading to inappropriate responses and eventually cyst formation. (**B**) Schematic representation of Hh-signaling in normal conditions with and without Hh-ligand present. Abnormal Hh-signaling in the absence of primary cilia or in retrograde dynein-2 mutants.

*Physical sensation* of flow, pressure, touch and vibration is referred to as mechanosensation. Although cilia are flexible, there is a basal level of rigidity provided by the axonemal microtubule network. Much of our understanding of mechanosensory mechanisms associated with cilia were derived from studies of renal tubules that are lined with ciliated epithelial cells, where the force of luminal fluid flow determines the direction and bending of the cilium (Figure 2A) [58]. The polycystin proteins PC1 and PC2 (encoded for by *PKD1* and *PKD2* genes which when mutated cause ADPKD) play a key role in mechanosensation and heterodimerize into an ion channel-complex [58]. The causal gene for the related pathology ARPKD, the *PKHD1* gene encoding fibrocystin, is associated with the polycystin complex [59]. Upon shear stress, the polycystin complex opens and imports extracellular Ca^2+^, elevating the intracellular Ca^2+^ concentration. Subsequently, modulated intracellular Ca^2+^ levels can act as a general second messenger to affect multiple downstream processes. Next to its role in mechanosensation, PC1 can additionally act as a transcriptional activator. In the presence of flow, PC1 is normally sequestered to the cilium; when flow is ceased, protealytically cleaved PC1 can, together with STAT6 and p100, activate a transcriptional program [60]. The relevance of polycystin function in adult tissue appears overall less dramatic compared to developmental stages, showing rather mild renal failure compared to the acute development of large cysts respectively [61].

The complexity of Ca^2+^ as a second messenger is complicated by excessive cross-talk between downstream targets and the large number of potentially affected targets. One important pathway downstream of mechanosensation-induced Ca^2+^-signaling is the Wnt-pathway. In unperturbed renal epithelial cells, for example, increased Ca^2+^ promotes the non-canonical over canonical signaling, whereas in various cilia-defective cells, an upregulation of canonical Wnt-signaling is noted [62]. The Wnt-signaling pathway is discussed in more detail below.

In other cell types, including sensory neurons, cilia bending also results in modified cAMP- signaling which can ultimately activate MAP/ERK-signaling and downstream proliferation [63]. In PKD, reduced intracellular Ca^2+^ concentration results in decreased phosphodiesterase 1-mediated conversion of cAMP to AMP, with consequent amplification of cAMP-signaling and downstream MAP/ERK target activation [62]. In turn, deregulation of the TSC1/2-mTOR pathway by cAMP/MAPK also associates with PKD; however the direct role of Ca^2+^ in mTOR-signaling has not been properly studied. For detailed reviews on cilia/Ca^2+^- signaling in the developments of renal cysts, we refer to comprehensive reviews by Abdul-Majeed and Nauli [62] and Kotsis, Boehlke and Kuehn [64]. Other regulators of mechanosensation, physical conditions such as osmotic pressure, heat shock, touch and extracellular matrix movements or vibrations can also excite ciliary-signaling and the mechanisms involved are slowly emerging [65,66].

*Hedgehog* (Hh)-signaling largely depends on cilia integrity. It was noted that inhibition of ciliogenesis in mouse knockout models of *kif3a* or *ift88* showed phenotypic overlap with mutant members of the hedgehog-signaling pathway [1]. Hh-signaling is implicated in many diverse (embryonic) developmental processes and includes regulation of tissue patterning and cellular differentiation, proliferation and survival [67]. Hh-signaling can be initiated by three ligands; sonic-Hh (Shh), Indian-Hh (Ihh) and Desert-Hh [68], the summarized mechanism described below is based on the best characterized member Shh. Although the exact mechanisms are not fully elucidated and there appears to be tissue-specificity, there is a basic understanding of how the pathway acts through the cilium (Figure 2B). In the absence of Hh-ligand, the hedgehog ligand binding receptor Patched (Ptc) localizes to the cilium and through an unknown mechanism Smoothened (Smo) is mostly retained in vesicles and excluded from localizing to the plasma membrane and entering the cilium [69], although a basal level is thought to traffic through cilia as well [70]. There are three mammalian paralogs of the Glioma family described; Gli1, a transcriptional activator that is upregulated after initial pathway activation, Gli2, the principal transcriptional activator and Gli3, the principal transcriptional repressor [71]. In the absence of Hh-ligand, Gli2 and Gli3 are complexed with the negative regulator Sufu and Kif7, which serves as a scaffold for PKA, GSK3β and CK1, promoting a cullin3/ubiquitin proteasomal-mediated cleavage that generates the Gli2R and Gli3R repressor forms, allowing the GliR’s to translocate to the nucleus and repress transcription [72]. In the presence of Hh-ligand, Ptc is excluded from the cilium and Smo becomes activated and translocates to the ciliary membrane, facilitated by β-arrestin and Kif3a and possibly other factors [71]. Simultaneously, Kif7, Sufu, Gli2 and Gli3 transition to the ciliary tip and the Sufu interaction is lost, allowing Gli2A and Gli3A stabilization. How Smo exactly activates GliA is not fully elucidated, but ciliary Smo is required for GliA formation, and probably does so by antagonizing Sufu. Although GliR regulation by Sufu does not require the presence of cilia [73], efficient formation of the repressor forms does not occur in cells without cilia [74]. Upon activation, GliA is dependent on retrograde IFT (dynein mediated) to exit the cilium after which it translocates to the nucleus to drive the expression of target genes. Defects in cilia integrity therefore modulate Hh-signaling, and depending on the underlying defect, either activate or dampen Hh-signaling [69]. Developmental processes that depend on hedgehog activity, such as neural tube patterning, are affected by defects in the pathway activation as is observed in cilia-deficient cells that fail to generate GliA. Alternatively, limb development requires efficient GliR formation, which is also perturbed in cilia-deficient cells [69]. Kif3a mutants, most IFT-B complex mutant alleles that perturb cilia formation (IFT52, 57, 88, 172), as well as other inhibitors of cilia formation such as TTK2, result in a constitutive dampening of the Shh pathway, as cilia are required for proper GliA and GliR processing [7,75,76]. Defects in the dynein-2 motor impede retrograde transport leading to an accumulation of proteins at the ciliary tip; consequently the membrane at the ciliary tip expands to form a bulge [77]. Dynein-2 mutations also impair Hh-activation due to the inability of GliA to translocate to the nucleus. Mutations affecting IFT-A complex members IFT139/TTC21B and IFT122, which primarily regulate retrograde axonemal transport, hyperactivate the pathway [78,79]. In contrast, other IFT-A mutants (IFT121, IFT144) display similar effects to IFT-B mutants, indicating that these are required for cilia formation, possibly reflecting the complexity of interplay between IFT-A members in transport of membrane proteins like Smo and ACIII [35]. For an extensive description of the relation between cilia and Hh-signaling, we recommend reviews by Goetz and Anderson [69] and Robbins *et al.*[71].

**Figure 3:**
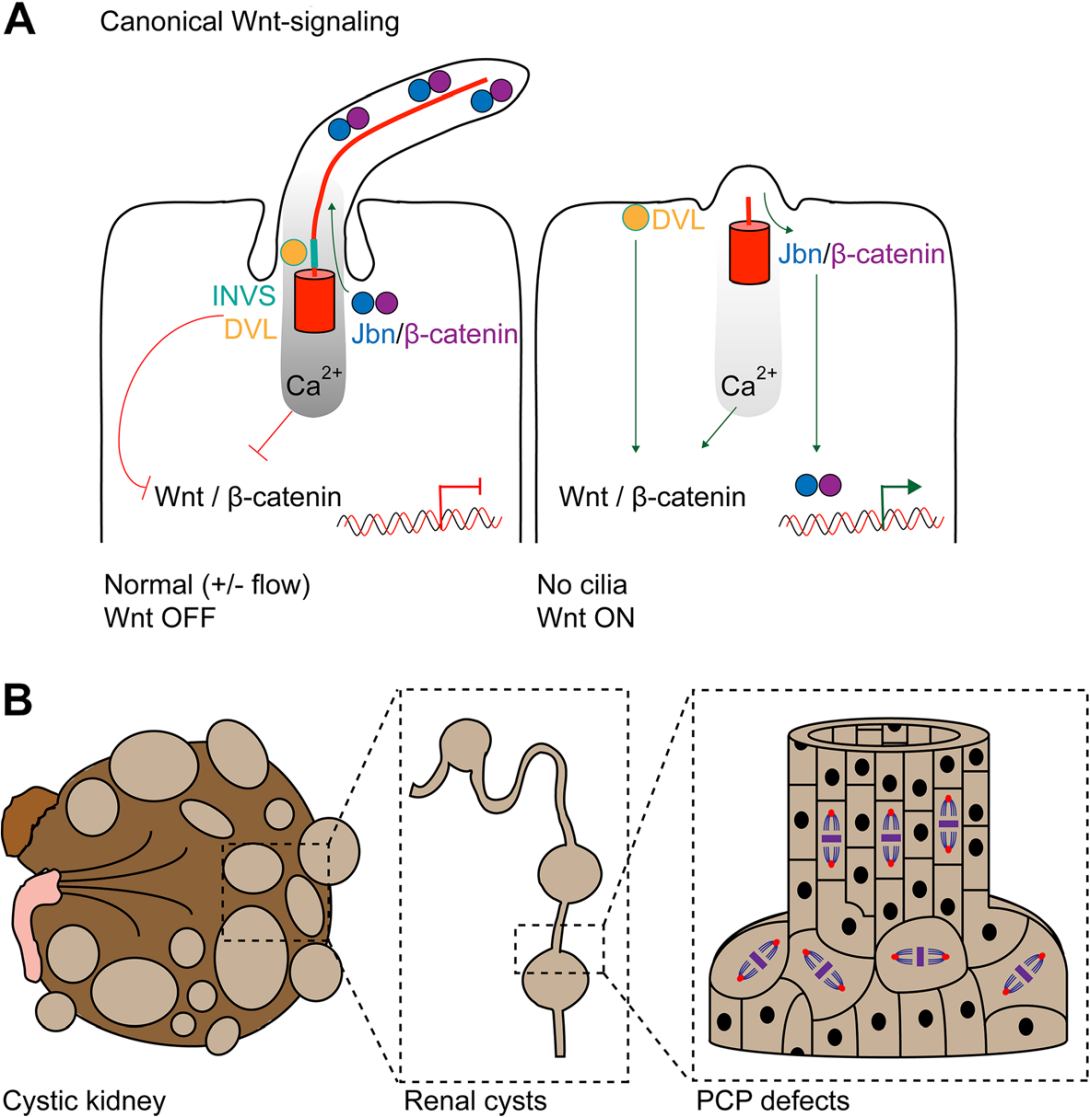
**Canonical and non-canonical/PCP-signaling and cilia. **(**A**) Canonical Wnt-signaling. When cilia are present, multiple mechanisms dampen Wnt-signaling; DVL is recruited by INVS/NPHP2 to the cilium, normal flow sensation elevates intracellular Ca^2+ ^levels that switch Wnt-signaling off, Jouberin (Jbn) sequesters a pool of β-catenin and recruits it to the ciliary compartment. In cilia mutants, mislocalized INVS fails to recruit DVL, which translocates to the membrane and activates Wnt-signaling. Ca^2+ ^response is lost, which fails to switch Wnt-signaling off. Jbn and β-catenin potentiate Wnt-signaling as a larger pool can translocate to the nucleus. (**B**) Illustrative model of cystic expansion of a renal tubule in a polycystic kidney. Disturbed PCP affects the orientation of cell division within the plane of tissue organization.

Both canonical and non-canonical *Wnt*-signaling regulate developmental and homeostatic processes [80]. The cilium seems to play a role in dictating the outcome of Wnt-ligand binding towards either pathway, but the details are not fully understood and data occasionally conflict. Put simplistically, in the absence of canonical Wnt-signaling, β-catenin is caught in a destruction complex together with Axin, adenomatous polyposis coli (APC), casein kinase 1 (CK1) and glycogen synthase kinase 3β (GSK3β) [80]. Wnt-ligand binding to the transmembrane receptors frizzled (Fz) and low density lipoprotein receptor-related protein 5/6 (LRP5/6), recruits dishevelled (Dvl) and Axin, resulting in disassembly of the destruction complex and subsequent accumulation of β-catenin in the nucleus, where it regulates transcription factors [80]. One body of evidence suggests a role for cilia in restraining canonical Wnt (Figure 3A). Downregulation of essential ciliogenesis genes *BBS1*, *BBS4*, *MKKS* or *KIF3A* in HEK293T cells and zebrafish embryos [81] as well as *Kif3a*, *Ift88*, *Ofd1* in murine cells [82] lead to nuclear β-catenin accumulation and enhanced transcriptional activity of canonical Wnt target genes. Similarly, genetic mutant *Chibby* mice [83] as well as zebrafish mutant for the *seahorse* allele [84], encoding cilia-associated (Chibby) and related (Seahorse) proteins, result in canonical Wnt activation. The nephronophthisis protein NPHP2/INVS normally recruits Dvl, thereby rendering β-catenin in the destruction complex; disruption of INVS allows Dvl to translocate to the membrane potentiating nuclear β-catenin accumulation [85,86]. Furthermore, nuclear β-catenin is a common hallmark of renal cysts, and oncogenic β-catenin is sufficient to drive cytogenesis [87]. The JBTS protein Jouberin/AHI1 also seems to inhibit canonical β-catenin-signaling via sequestration to the primary cilium; AHI1 regulates both β-catenin nuclear import and ciliary localization. Defects in cilia formation potentiate Wnt responsiveness, and tethering β-catenin to the cilium reduces canonical Wnt-signaling [88]. In line with this notion, Ahi mutant mice do not show nuclear β-catenin accumulation and signaling in their kidney cysts, which also raises the question of how relevant canonical Wnt-signaling is for renal cyst development [89]. Several other studies similarly contradict the activation of Wnt-signaling solely due to cilia disruption; *Ift88*, *Ift172* and *Kif3a* mutant mouse embryos and maternal-zygotic zebrafish *ift88* mutants do not show altered canonical Wnt-signaling [90,91], suggesting that results reflect tissue and developmental stage-specific discrepancies [69]. Another study indicated that altered Wnt-signaling is a secondary defect of modified Hh activity [92]. Importantly, Ca^2+^ levels can also affect the canonical Wnt pathway [93], and Ca^2+^ influx as a result of mechanosensation is proposed to switch canonical Wnt off, whilst turning non-canonical signaling on [94]. The absence of proper flow-sensing in organs with dysfunctional cilia impairs Ca^2+^-mediated Wnt regulation which further complicates dissection of the various pathogenic pathways. Likely Wnt regulation occurs through various mechanisms, even in close proximity to the cilium and basal body and the effect of cilia towards canonical Wnt-signaling is subtle, dependent on cell type and developmental stage. Generally, defects associated with defective cilia/β-catenin-signaling appear less severe compared to Hh- mediated-signaling [95].

In the non-canonical pathway, β-catenin is dispensable and Wnt-signals act under control of planar cell polarity (PCP), but again there are conflicting data on the direct role of cilia in PCP-signaling. PCP regulates the correct expansion and homeostasis of polarized tissue (Figure 3B) and it is evident that many ciliopathies feature PCP defects; for example, the cystic phenotypes are likely a defect of altered PCP [50]. Convergent extension during gastrulation in development is a PCP-mediated process, and defects in cilia genes such as *INVS*, *BBS1*, *BBS4*, *BBS8*, *MKKS* and *OFD1* induce convergent extension defects [95]. These data collectively point to the hypothesis that cilia are involved in the switch between canonical and non-canonical Wnt-signaling, which is independent of the PCP-signaling pathway [95]. A developing view in the field is that cilia are not extensively involved in regulation of polarity proteins through deregulating PCP-signaling, but that this defective polarity is the result of mis-positioning of the centrosome during cell division [95]. Indeed, many ciliopathy proteins such as IFT88, OFD1 and BBS4 are essential for proper centrosome composition and structural stabilization [96-98]. PCP-signaling itself can affect correct basal body docking and ciliogenesis [99]. Studies in mammalian cells are sparse however, and most knowledge has been obtained from *Drosophila* and *Xenopus* studies. Essential PCP- signaling molecules that affect ciliogenesis include Fuzzy, Inturned, Fritz and Dvl. The molecular role for these proteins is emerging and it is suggested they affect processes including actin remodeling and ciliary-vesicle transport [95]. For a more extensive discussion about the relation between cilia, Wnt-signaling and PCP we recommend a review by Wallingford [95].

*Platelet-derived growth factor* (PDGF)-signaling affects cell migration, proliferation and survival [100]. Upon cell cycle exit, the receptor PDGFRα is upregulated and localizes to primary cilia in cultured fibroblasts [101]. Binding of the ligand PDGF-AA activates the dimerized PDGFRαα receptor and downstream Akt, ERK1/2- and MEK1/2-signaling molecules [101]. Defects in primary cilia formation disrupts PDGF-AA-signaling and affects endothelial cell function [102]. The PDGF receptor is a receptor tyrosine kinase (RTK); recently other RTK-signaling events were described as being mediated through primary cilia in some cell culture models, including epidermal growth factor receptor (EGFR), insulin-like growth factor (IGF1R), and the angiopoietin receptor (Tie-2), For a detailed description of the implications RTK-signaling might have in light of cilia sensing, we refer to a recent review by Christensen, Clement, Satir and Pedersen [103]. Fibroblast growth factor (FGF)- signaling has been shown to affect cilia length and affect left-right determination, a process dependent on proper cilia functioning [104].

*Hippo-*signaling (Salvador-Warts-Hippo) has recently been added to the growing list of signaling pathways at least partly regulated through the cilium. Hippo-signaling is based on a number of serine/threonine-kinases that are involved in controlling organ size and cell proliferation. Many members act as tumor suppressor proteins as well as proto-oncogenes [105]. NPHP4 interacts with the Hippo regulator LATS, allowing the transcription factors YAP and TAZ to translocate to the nucleus. In the absence of NPHP4, Hippo-signaling is overactive and cell proliferation is limited. NPHP4 may regulate the renal fibrosis associated with most ciliopathies through CTGF transcriptional regulation [106]. The Crumbs receptor family is known to affect Hippo-signaling [107] and Crumb3 has been shown to localize to the cilium [108]. Furthermore, Hippo pathway core component Mob1 delays ciliogenesis in *Tetrahymena*[109]. Histological examination of human PKD sections demonstrates nuclear translocation of YAP and TAZ [110], and deregulated Hippo-signaling itself can result in cyst formation, possibly through cross-talk between Hippo- and canonical and non-canonical Wnt-signaling [94].

Apart from these core-signaling pathways receiving much attention in the past years, new developments have expanded the list of cilia-related-signaling. In endothelial cells, endothelial to mesenchymal differentiation depends on cilia function towards TGFβ activity [111]. In skin development, cilia are required for proper Notch-signaling and progenitor cell differentiation [112]. Tubby proteins were recently shown to serve a bridging function between specific membrane domains and IFT, affecting signaling [113], and alterations of cilia membrane composition itself are sufficient to disrupt signaling [114,115]. The coming years will likely show an upsurge of signaling modulation regulated by the primary cilium.

### Reciprocal regulation of cilia and the cell cycle

**Figure 4:**
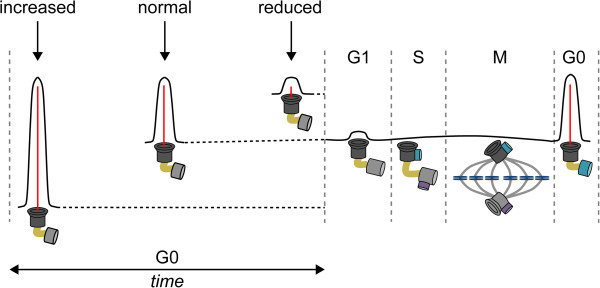
**Cilia length in control of cell cycle progression. **Cilia provide a physical block for cell cycle progression by laying claim to the basal body. Disassembly of the primary cilium is required to liberate the centrosome and allow duplication during S-phase and subsequent formation of the mitotic spindle during chromosomal segregation. Cilia mutants that inhibit ciliogenesis are prone to initiate rapid cell duplication when properly stimulated, and in contrast, increased axonemal length delays cell cycle progression. The maturation steps (growth and appendages) of the centrioles are indicated during the various cell cycle stages.

The eukaryotic cell cycle dictates and regulates cellular duplication, and recognizes five consecutive but distinct phases. The basal stage is referred to as interphase or G1. The G1 centrosome has one mature or mother centriole that is equipped with distal appendages and one incomplete daughter centriole. Once cells are properly stimulated by mitogenic or growth factors they prepare for entry into S-phase, and towards the end of G1-phase the daughter centriole matures to a full-length centriole. When cells enter S-phase, the chromosomes and the centrosome are duplicated and both centrioles serve as a platform to assemble two new incomplete centrioles; master regulators driving centrosome duplication include PLK4 and SAS6 [116]. Following S-phase, cells enter G2 to prepare for the physical chromosome partition; the initial mother and daughter centrioles together with the newly formed pro-centrioles separate and migrate to opposing sides. After nuclear envelope breakdown and chromosome condensation, the centrosomes become a regulatory center for bipolar spindle formation that connects microtubules to the chromosomes. During metaphase, all chromosome pairs must be connected to either centrosome and congress to the metaphase plate. Once the spindle assembly checkpoint is inactivated cells proceed into anaphase, the duplicated chromosomes are disengaged and exactly one copy of each moves apart towards the centrosome. The site of the metaphase plane now becomes a site where the membrane is progressively invaginated. The remaining microtubules allow membrane vesicles to accumulate and deposit membrane and other essential components of the cytokinetic machinery. When the remaining cleavage furrow is broken, cells pinch off from each other and return to G1. During late G2/M, the daughter centriole has matured to a mother centriole with the addition of distal appendages. After cell division, each cell contains one new centrosome composed of either a grandmother or mother centriole and both contain one incomplete daughter centriole [116]. Most cells in the human body will enter the G0 or quiescent stage after successful cell division (Figure 4). Because the centrosome is involved in both cell division and ciliogenesis, these processes are mutually exclusive and there is a continuous tipping of balance to recruit the centrosome for either process. Upon entrance into G0, the mother centrioles have the unique capacity to dock to the cell membrane and initiate axonemal growth, as discussed above. An exception to the rule are multiciliated cells; in these terminally-differentiated cells, centrosomes must be assembled *de novo* and one cell can form up to hundreds of centrosomes, each having the capacity to form a cilium [116].

A number of proteins intimately associated with ciliogenesis and cilia function are additionally implicated in cell cycle control, which can be achieved by functioning within any of the numerous essential cell cycle processes described above. We will first discuss the cell cycle defects observed for a number of cilia proteins that can result in alterations in cell cycle timing and the fidelity of centrosome duplication, chromosome segregation and cytokinesis. Microtubule-anchoring to the centrosome requires functional BBS4, which interacts with the microtubule network organizer PCM1. Knockdown of BBS4 in cell culture blocks cell division progression and induces an increase in apoptosis [98]. Bbs4 knockout mice similarly show disorganized microtubules in dendrites, but no severe apoptosis was described, suggesting tissue-specificity of the BBS4/PCM1 interaction and redundancy *in vivo*[117]. A similar mouse knockout phenotype [118] and defect in cytokinesis has been shown for MKKS/BBS6 upon cell culture knockdown, however in contrast to the BBS4 phenotype, no defects in microtubule organization were observed; the authors suggested that the chaperonin activity of BBS6 might be required for a cytokinetic-specific process [119]. IFT88 overexpression in non-ciliated cells blocks G1/S transition and depletion induces cell cycle progression [120]. IFT88 interacts with the Rb inhibitor Che-1, suggesting a model in which IFT88 depletion allows Rb to become active and induce cell cycle progression independent of a microtubule and dynein interaction [120]. The ORPK/*Ift88* mouse mutant accordingly displays epithelial cell hyperproliferation in several tissues [121,122], but it remains undetermined if this directly attributable to the IFT88/Che-1 interaction, as ciliary- signaling through Wnt, for example, is also modified. Another IFT-B complex member, IFT27, delays cell cycle progression upon knockdown in *Chlamydomonas reinhardtii* and affects cytokinesis [123]. Apart from associating with the IFT-B complex, stoichiometric data suggests about half of the IFT27 protein does not interact with this complex. Given that IFT27 is a Rab-like G-protein, it has been suggested that IFT27 might have a transport function from the centrosome to cleavage furrow during cytokinesis [123]. Interestingly, knockdown of IFT27 also resulted in reduced expression of other IFT members, including IFT46, IFT52, IFT81 and IFT139. Another study showed that the IFT27 and IFT46 are dynamically regulated during the *Chlamydomonas* cell cycle, peaking during the S-M cell division phases, indicating that transcriptional control of these IFT members is restricted within the cell cycle [124]. Furthermore, IFT27, IFT46, IFT72, IFT139 relocalize to the cleavage furrow during cytokinesis [124]. Together with the notion that IFT proteins play roles in non-flagellar transport, such as during the formation of the immunological synapse in T-cells [44], it could now be suggested that some IFT components play general roles in membrane transport, not limited to IFT [124]. This is further supported by the evolutionary origins of core IFT members that share homology to other transport coat complexes such as COPI [125]. This notion is supported by another study that described a large overlap in the cellular machinery to regulate cytokinesis and ciliation by comparative proteomics and was more extensively shown for PRC1, MKLP-1, INCENP and centriolin in *Caenorhabditis elegans* and cell culture studies [126].

One proposed explanation for the cell cycle defects observed is that the balance between centrosome and basal body transition is disturbed, leading to cell cycle checkpoint activation [127]. This seems to be accompanied by activation of key regulators of the G1/S transition, Cdk2 and cyclin E, when centrosomes are disengaged from ciliation [127]. Alternatively, proteins that are required for centrosome biogenesis and whose absence leads to structural defects, induce a p38/p53/p21-dependent checkpoint activation [128]. Overall, evidence is accumulating that a number of components initially linked to the cilium have functions that are not limited to the cilium alone, but have retained or acquired properties that render them essential to alternative cellular processes. These could either be through evolutionary conservation and a specificity that is less stringent towards the cilium than expected or dual functions of a particular protein.

The mechanisms controlling ciliogenesis are discussed in more detail above, but one of the first prerequisite is the absence of proliferative stimuli; *in vitro*, most cell lines require serum deprivation to enter G0 and initiate ciliogenesis. Another factor is ascertaining established polarity. Inversely, there are also mechanisms controlling ciliary disassembly, which are incompletely understood. Depending on the cell type, disassembly occurs in S-phase or before the G2/M transition [129], and is known to be initiated in two waves [130]. The best-studied disassembly mechanism involves the Aurora A kinase, which can be activated by the scaffolding protein HEF1/NEDD9. Aurora A in turn activates the tubulin deacetylase HDAC6, hence destabilizing axonemal microtubules and initiating cilia resorption [130]. *Pitchfork/PIFO* localizes to vesicles and the basal body and it can similarly activate Aurora A through direct interaction. Reducing PIFO in murine embryos generates mitotically arrested cells with ciliated spindles, whereas overexpression generates centrosomal overduplication [131]. Alternatively, HEF1 can be activated by canonical Wnt-signaling components Wnt3a, Dvl2 and β-catenin [132]. In addition, activation of the non-canonical Wnt pathway through Wnt5a and casein kinase-1-epsilon-induced complex formation of Dvl2 and Plk1, that stabilizes HEF1 and allows Aurora A activation to induce cilia disassembly [133]. The never in mitosis A (NimA) related kinase NEK2 is another important mediator essential for cilia resorption at the G2/M transition, but the exact mechanisms involved and a connection to the Aurora A pathway are uncertain [134]. In contrast to controlling timely resorption of cilia, other networks of centriolar proteins prevent aberrant cilia assembly. CP110 localizes to the distal end of centrioles where it interacts with, and prevents, NPHP6 and Rab8a from initiating ciliogenesis [135]. A distinct complex of CP110 and CEP97 also prevents cilia assembly [136]. In quiescent cells, both KIF24 and its interaction partners CP110 and CEP97 are removed from the mother centriole distal end to promote ciliogenesis [6]. KIF24 depolymerizes centriolar microtubules to prevent premature cilia assembly, and loss of KIF24 promotes ciliogenesis even in cycling cells [6]. The recently described protein TTBK2 promotes the removal of CP110 to allow for cilia formation [7]. Furthermore, a CP110/Centrin/CaM module regulates cytokinesis [137], indicating a broad functional spectrum for CP110. The complex interplay of regulators of correct cilia/centriolar size eventually affects the crossroad of basal body and centrosome.

It is becoming clear that the axonemal length directly influences cell cycle time (Figure 4) and reduction of cilia length or cilia depletion allows cells to enter S-phase more rapidly. Mutations in *INPP5E* (also called JBTS1) disrupt the balance of phosphoinositides which makes cells more prone to re-enter the cell cycle upon growth factor stimulation; functional *in vitro* studies suggest the cilium can dampen the response to mitogenic cues and prevent premature cell cycle entry [114,115]. INPP5E interacting proteins CEP164, ARL13B and PDE6 are required for INPP5E targeting to the cilium with depletion of these factors associating with the severe JBTS and MKS ciliopathy syndromes [138]. Inversely, increased cilia length can delay cell cycle re-entry. Recently, it became evident that Nde1 and Tctex1 regulation of dynein subunits affects cell cycle progression [139]. Nde1, which is normally expressed at low levels in quiescence, recruits axonemal dynein complex member LC8 and subsequent cilia length suppression. Depletion of Nde1 results in increased ciliary length, thereby delaying cell cycle progression [140]. Tctex1 associates with the cytoplasmic dynein complex, but upon activation relocates to the basal body where it promotes ciliary disassembly and cell cycle progression [141]. Previously identified factors that increase cilia length are male associated germ-cell kinase (MAK) and cell cycle-related kinase (CCRK) [13]. TSC1 and TSC2 similarly increase cilia length in response to energy/nutrient sensing (discussed further below). Intracellular levels of second messengers also influence cilia length; lowering Ca^2+^ and elevating cAMP levels increases cilia length. Under flow conditions, intracellular Ca^2+^ levels rise again and cAMP decreases, reducing cilia length [142]. Phosphatase inhibitor-2 (I-2) is necessary for microtubule acetylation and thus stability, and is located on the membrane in proximity to the docked basal body. Inhibition or deletion of I-2 reduces cilia length [143]. Several ciliopathy syndromes are associated with neurological disorders, suggesting that cilia might be involved in the development of these diseases. A recent RNA interference screen in NIH3T3 cells, targeting selected genes that were previously identified in genome-wide association studies for neurological disorders, indeed appeared to affect cilia length negatively. In contrast, three targets significantly enhanced cilia length; CCDC18, FOXP1 and MIR137, although the underlying mechanism was not determined [54]. Also, G-protein coupled receptors are frequently located to primary cilia, such as serotonin and somatostatin, as well as the recently described shh antagonist Gpr161 [31]. Some G-protein coupled receptors affect cilia length; activation of dopamine D5 receptor increases cilia length [144], as does dopamine receptor D1 [145]. Modification of cilia length might also be important for signal interpretation, for example in the CNS.

### Cilia and cancer

Because cilia have the ability to physically influence the cell cycle and manipulate signaling cascades, it has been a long-running hypothesis that defective cilia biogenesis could be an important step in cancer development. However, much of the data in this arena rests on *in vitro* studies and it thus remains to be established how, and if, there is a direct relationship between ciliary dysfunction and tumorigenesis. One of the major arguments against a direct role of cilia in tumorigenesis is the lack of evident tumor predisposition in patients with many classic ciliopathies such as SLNS, LCA and NPHP. However, mortality from organ function impairment may mask true tumor incidence in ciliopathy patients. The JBTS disease gene *JBN/AHI1* sensitizes tissue for Wnt activation, allowing low Wnt levels to improperly activate cells and induce over-proliferation. Accordingly, oral hamartomas have been associated with Joubert Syndrome-related disorders [146,147]. There is some conflicting data on the risk of heterozygote relatives of BBS patients to develop renal cancer; one small study recognized a 17-fold increase risk [148], but a larger study disputed this finding in their cohort [149]. To date though, with the exception of a few case reports, cancer has not been reported to associate systemically with the relatives of these recessive ciliopathies. Heterozygous carriers therefore do not seem to be at increased risk for cancer development, but it will be important to closely monitor these carriers in respect to tumor development and determine putative biallelic inactivation. Impaired cilia function can induce inappropriate responses in progenitor cells, expanding the stem cell compartment or differentiate into dysplastic tissue, as is observed in the epidermis, vascular system and mammary gland [111,112,150]. Alternatively, it is becoming evident that many proteins encoded for by familial cancer genes do actually affect cilia function. The cilium is an important mediator of homeostasis and a growing number of proteins which affect both cell ciliation and tumorigenesis have been identified.

### Clinical observations of cilia and cancer

As most tissues in the human body at least have the capacity to express cilia, it is important to address the effect on cilia expression in corresponding tumor types. Currently this is still an understudied field, and only a small number of tumor types have been subjected to detailed cilia analysis [151]. In breast cancer development there is little information on cilia involvement available, however, the cilia-associated genes *Gli1* (Hh effector), *RPGRIP1* (LCA) and *DNAH9* (PCD) are commonly mutated in breast cancer [152,153]. A later study indicated that ciliary frequencies are decreased in breast cancer tissue and breast cancer-derived cell lines when compared to normal breast tissue fibroblast and epithelia; cilia frequencies after prolonged serum starvation were more severely reduced in cell lines derived from aggressive cancer lines, furthermore this was shown to be independent of increased proliferation through determining the number of Ki67 positive cells [154]. Accordingly, analysis of murine mammary glands indicates that cilia are expressed during development and remain present on myoepithelial and stromal cells but are absent from luminal epithelia in matured mammary glands [155]. Detailed analysis of Hh-signaling and primary cilia in mammary basal cell hyperplasia characterizes the Hh-responsive cells as progenitor-derived cells bearing cilia [150]. The proliferation rates of these cilia-bearing cells is lower compared to control tissue [150]. Furthermore, it was shown that *NPHP9* (*NEK8*), which is upregulated in breast cancer [156], modulates cilia length and activates the oncogenic Hippo pathway transcription factor TAZ [157]. Melanoma development has several stages, ranging from melanoma *in situ*, primary invasive melanoma and metastatic melanoma. While melanocytes express primary cilia, early melanoma *in situ* express hardly any cilia, and cilia are completely lost in progressive tumor phases. In this study, proliferation rates as determined by Ki67 staining were typically too low to account for the observed reduction of cilia frequency [158]. Like melanoma, pancreatic ductal adenocarcinoma is also characterized by activated Ras-signaling [158], and similarly reduced ciliary frequencies were observed in early stages of tumor development [41,159]. A loss of cilia was also observed in ovarian cancer originating from the ovarian surface epithelium that were growth arrested to normalize for the effect of proliferation on cilia expression [160]. Another study of an ovarian serous cystadenoma, based on co-evolutionary analyses, identified an overrepresentation of mutated cilia genes in this tissue [161]. On a more general note, co-evolutionary analyses revealed that the cilia proteome (ciliome) evolved concomitantly with multi-cellularity and adopted important functions in the regulation of cell-division control. Therefore, these authors postulate by extrapolation that deregulation of the ciliary network of proteins will result in proliferation in cancer development [161]. We recently published data suggesting a predisposition for tumor development in zebrafish that carry a heterozygous mutation in cilia gene *lrrc50* that also causes PCD [162]. Tumors isolated from these zebrafish are analogous to human seminoma, a subtype of the group of testicular germ cell tumors (TGCT), and germline loss-of-function mutations were identified in human seminomas from patients with a family history of seminoma formation. It is unclear, however, if mutant *LRRC50* tumor formation is attributable to impaired cilia function but the mutations found in the seminomas demonstrate cilia-associated gastrulation defects in fish embryos [162]. Colorectal cancer is increasingly associated with a role for cilia. The cilia disassembly promoting kinase *Aurora A* is often mutated in colorectal cancer [163], as well as Hh member *Gli3* and polycystic kidney disease-causing *ARPKD1*[164]. Elegant murine studies have unraveled the role of cilia and crosstalk with the Hh-signaling pathway in the development of skin tumors of the basal cell carcinoma (BCC) subtype [165] and subtypes of medulloblastoma brain tumors [166]. These studies collectively demonstrate *in vivo* that cilia can either promote or repress tumor formation, but depletion of cilia formation alone is not sufficient to drive tumor formation, and tumor development requires driving oncogenic mutation of Hh-components [167]. BCC, medulloblastoma and other cancers, including pancreatic and ovarian cancers described above, often upregulate Hh-signaling. In tumors driven by upstream mutations in the Hh pathway (Ptc, Smo, Sufu or excessive Hh-ligand), the cilium is required to process GliA and activate downstream targets [151]. Intriguingly, in mutants driven by oncogenic Smo, simultaneous blocking of cilia formation by knocking out *Kif3a* or *Ift88*, resulted in impaired GliA formation and inhibition of tumorigenesis, suggesting that in this model, tumor formation is cilia-dependent [165,166]. If tumor formation is caused by mutations downstream of cilia (Gli1A, Gli2A), cilia are obsolete for Hh activation. However, restored ciliation dampens Hh-signaling, which is explained by the formation of Gli3R that counteracts GliA [151]. Thus, selection for the presence or absence of cilia of these cancer types entirely depends on the underlying oncogenic mutation. Medulloblastomas featuring oncogenic mutations in β-catenin also retain cilia, indicating that cilia function is still required for tumor formation. Although cilia generally reduce β-catenin nuclear-signaling, which would place selective pressure for cilia-loss in tumors, it is possible that a threshold level of Hh-signaling is still required in some tumor types, leading to cilia retention [166]. On a more general note, tumors that depend on cilia retention seem to have better prognosis with regard to survival [166]. In addition to these cell-autonomous Hh effects, increased Hh-signaling through autocrine or paracrine signals from the tumor stroma adds another layer of complexity [168].

Cilia loss is prevalent in various forms of kidney cancer. Renal cysts are a hallmark phenotype of cilia dysfunction and prevalent in many classic ciliopathy syndromes [49]. Accumulating evidence suggests that renal cystic lesions are a precursor stage of tumor formation; patients with acquired cystic kidney disease (ACKD) have an increased risk for renal cancer with incidences ranging from 2 to 5% [169]. It was recently demonstrated that chronic lithium therapy (a standard therapy for bipolar disorder) increased the risk for ACKD and kidney cancer development; lithium activates GSK3β by phosphorylation, resulting in increased β-catenin-signaling, which is known to suffice for renal cyst formation [87,170]. Renal cell carcinoma (RCC) can be subdivided into clear cell RCC (ccRCC), chromophobe (chrRCC) and papillary RCC (pRCC) subtypes [171,172]. Renal oncocytoma closely resembles the chrRCC pathology [173,174], and both tumor types arise from unciliated intercalated cells of the renal collecting duct. Analysis of a small cohort of ccRCC indicated severely reduced cilia frequencies and a more subtle reduction in pRCC [175], our own results, in a large group of ccRCC compared to parenchymal tissue confirmed this and also indicated reduced frequencies in oncocytomas and chrRCC [176]. Both studies excluded the possible confounder that the fewer cilia observed were due to increased cell proliferation through scoring for Ki67-positive cells [175,176]. Intriguingly, cancer syndromes that predispose to RCC development, encoded for by mutant *VHL*, *FLCN* or *TSC1/2* (discussed in greater detail in a separate section), all have been shown to assert molecular activity towards cilia function and structure in addition to their previously recognized functions in relaying responses to metabolic factors. This puts forward the hypothesis that kidney cancer is a two-step process in which both primary cilium function needs to be impaired as well as modification of metabolic pathways; uncoupling regulation by energy, nutrients, oxygen and iron. Of interest, in a family predisposed to RCC and thyroid cancer, a genomic breakpoint was identified disrupting TRC8, a protein related to PTCH [177].

### Familial cancer syndromes and cilia

Genetic predisposition to solid tumor development is typically associated with heterozygous germline mutations in tumor suppressor genes or oncogenes. Identifying the genes and pathways mutated in these rare cancer syndromes has been proven remarkably useful in understanding early events of tumor development. Because a single patient with a germline mutation may have several lesions of varying degrees, it is possible to understand the genetic and cellular contribution to the natural course of that particular tumor type. Functional aspects of many of these genes have been elucidated, and interestingly, a number of well- established classic tumor suppressor proteins are involved in ciliary biogenesis, in addition to their attributed functions. The fundamental processes underlying cancer development and the tissues targeted in these syndromes are likely to require modulation of multiple cellular processes prior to carcinogenesis. Yet, the association of several classic tumor suppressor proteins and cilia suggests that ciliary loss, at least in some tissues like perhaps the kidney, can be associated with very early events in tumorigenesis.

For example, mutations in the *VHL* tumor suppressor cause the autosomal dominant familial cancer syndrome von Hippel-Lindau disease (VHL) that is characterized by the development of renal and pancreatic cysts, ccRCC, pheochromocytoma (tumor of the adrenal gland), cysts and hemangiomas in the central nervous system, retinal angiomas, and low-grade adenocarcinomas of the temporal bone [178]. In addition, *VHL* is also inactivated in up to 87% of sporadic clear cell RCC’s [179]. *VHL* loss conforms to the Knudson 2-hit model that dictates that both wild-type alleles of a tumor suppressor gene must be inactivated prior to tumor outgrowth [180]. Depending on the germline mutation resulting in complete loss-of-function or missense hypomorphic mutations, the disease is manifested in a subset or combination of target organs. VHL’s biological activity is complex; one major role is the degradation of HIF transcription factors, which become stabilized in hypoxic conditions and regulate the activation of a transcriptional program that promotes angiogenesis [164]. Given that artificially high activity of HIFs is not sufficient for RCC development [181], VHL must exert alternative protein functions. HIF-independent functions of VHL include regulation of cell polarity through Par3, Par6 and stabilization and orientation of microtubules [182]. Interestingly, VHL binds kinesin-2 subunits KIF3A and KAP, and it facilitates renal cilia mechanosensation [164]. Moreover, VHL seems to act in conjunction with GSK3β to maintain cilia; depletion of either protein alone does not affect ciliation, but activation of GSK3β in the absence of VHL initiates cilia disassembly [183]. The current view is that loss of VHL is sufficient for renal cyst formation, but that additional genetic lesions are required for subsequent degeneration of a renal cyst into ccRCC [164,180]. The observation that mice with inactivated *Vhlh* and *Pten* develop epididymal cystadenomas supports this model [184]. The chance of acquiring additional inactivating alleles at other loci is promoted by *VHL* loss, given that loss of VHL also reduces Mad2 levels, a protein that is part of the spindle checkpoint [185]. Aneuploidy and chromosomal instability drive subsequent genetic changes associated with RCC.

Tuberous sclerosis (TSC) is an autosomal dominant disorder that associates with neurologic disorders such as epilepsy, mental retardation and autism, but is mainly a tumor suppressor gene syndrome associated with renal cyst formation and tumorigenesis in various organs including the kidney, brain, retina and skin [186,187]. TSC is associated with germline mutations in *TSC1/Harmatin* and *TSC2/Tuburin*[188]. Renal phenotypes in TSC patients mostly comprise benign renal angiomyolipoma (50 to 80%), but a minority develops ccRCC (3%) [169]. Although the overall ccRCC incidence is only marginally increased compared to the general population, the onset of ccRCC development in TSC patients seems to occur earlier in life and other RCC pathologies are sometimes also observed [187]. Mouse models similarly develop cysts and RCC [189-191]. TSC disease manifestation requires genetic inactivation of the second allele of either *TSC1* or *TSC2*, rendering it a classic tumor suppressor gene. In contrast to VHL, loss of *Tsc1* or *Tsc2* enhances cilia length in mouse MEF’s and zebrafish mutants [186,192], which could explain why TSC patients clinically exhibit few renal cysts and relatively low RCC frequency [188]. The increased cilia length fits with the finding that disruption of a number of neurologic disorder-associated genes similarly enhance cilia length [54]. The mTOR pathway senses nutrient and energy levels and regulates cell growth accordingly. TSC1 and TSC2 normally form a heterodimer TSC1/2 that inhibits the TORC1 complex. Blocking the mTORC1 complex using rapamycin reduces cilia length, suggestive of a role for nutrient/energy-sensing in cilia length control [193]. The TORC1 complex functions upstream of the S6 kinase 1 (S6K1) which regulates ribosomal function and translation. Overexpression of *s6k1* in zebrafish also increases cilia length, making it likely that the TORC1 complex regulates cilia length through S6K1. Thus, loss of TSC1/2 releases the repression of TORC1, activating S6K1 and increasing cilia length [193]. In addition, GSK3β was found to function as an upstream activator of TORC1 [193]. The emerging view thus suggests that low energy/nutrient levels activates the mTOR pathway and via S6K1 enhances cilia length, which physically delays the cell cycle in a similar fashion to Nde1 and Tctex1 [140,141].

Functioning in the same nutrient/energy-sensing pathway is FLCN, which is the gene product of the disease locus for the monogenic disorder Birt-Hogg-Dubé (BHD) syndrome [194]. BHD syndrome is characterized by the development of renal cysts, kidney tumors of various histopathological subtypes, pulmonary cysts and benign cutaneous tumors (fibrofolliculomas) [195]. Renal tumors isolated from BHD patients are histologically diverse, predominantly consisting of chrRCC (34%) and hybrid oncocytoma/chromophobe (50%) neoplasms (both derived from intercalated cells of the collecting duct), and less frequently ccRCC (9%) [196]. Accordingly, mouse mutants for BHD-syndrome form renal cysts and RCC [197]. We have shown that FLCN levels affect ciliation [195,198]. In contrast to TSC1/2, FLCN does not affect cilia length but rather regulates the timing of ciliation. FLCN-associated cilia loss results in increased β-catenin-signaling and loss of FLCN additionally-induced PCP defects, which could explain the renal cystic phenotype in BHD patients [195,198]. FLCN has further been described to signal to AMPK, TSC1/2 and TORC1, however there is conflicting data and it remains uncertain whether FLCN activates or inhibits the mTOR pathway. In mouse renal tumors, loss of FLCN activates TSC1/2 [199]. It has been suggested that inappropriately high as well as low levels of TORC can lead to renal tumor formation [200], however, the precise role of ciliary-signaling in these events remains elusive.

Another member of the energy/nutrient-sensing pathway is the kinase LKB1, encoded for by *STK11*. Mutations in *STK11* predispose to the Peutz-Jeghers tumor syndrome (PJS) that features benign gastrointestinal (GI) polyps as well as malignant tumors in the GI, breast and gynecological organs. Somatic *STK11* mutations have been identified in lung, bladder and cervical cancer [201]. LKB1 is a master regulator of numerous downstream kinases, including AMPK and is upstream of mTOR-signaling [201]. Simultaneous inactivation of *lkb1* and *vhl* in zebrafish, however, neutralizes AMPK activation and the *lkb1* phenotype, suggesting a complex interplay between hypoxia and AMPK pathways [202]. The role of cilia in LKB1 regulation is currently limited, however it has been shown that cilia mechanosensation under flow conditions requires ciliary-localized Lkb1 to activate AMPK and mTOR-signaling to regulate cell size. This effect is independent of Ca^2+^-mediated mechanosensation [203]. Similar to FLCN and VHL, LKB1 and IFT88 independently regulate spindle positioning in epithelial cells [96,185,204].

Mosaic variegated aneuploidy syndrome (MVA) is a rare autosomal recessive childhood cancer disorder characterized by mosaic aneuploidies that give rise to rhabdomyosarcoma, Wilms’ tumor/polycystic nephroblastoma and leukemia [205]. Associated pathologies overlap with the ciliopathy disease spectrum; polycystic kidneys, microcephaly, Dandy-Walker complex, intrauterine growth retardation, mental retardation, infantile obesity, congenital abnormalities, eye abnormalities, postcerebellar cyst and hypoplasia of the cerebellar vermis [206,207]. MVA can be caused by biallelic as well as monoallelic mutations in *BUB1B* and *CEP57*[205]. *BUB1B* encodes for the spindle checkpoint protein BUBR1, defects in which cause premature chromosome segregation errors and neuploidy *in vitro*[208]. A recently identified additional function for BUBR1 is the regulation of ciliogenesis. Quiescence is maintained through proteasomal degradation of a number of substrates including DVL. Basal DVL activity is required for basal body docking and ciliogenesis, however, increased activity leads to downstream canonical Wnt-activation and cell cycle progression. In control and MVA patient-derived fibroblasts, BUBR1 was identified as an essential co-activator of the APC/C^CDC20^ complex that targets CDC20 for degradation and allows the APC/C^CDH1^ to maintain low DVL levels during quiescence through proteasomal degradation. Consequently, high levels of DVL in MVA-cells inhibit ciliogenesis. The inability of BUBR1 to bind CDC20 and inhibit APC/C^CDC20^ also disturbs the spindle assembly checkpoint and subsequent faithful chromosome segregation [207].

Basal cell nevus syndrome or Gorlin syndrome is a rare autosomal dominant disorder with an estimated incidence of 1 in 57,000, and is characterized by a variable disease spectrum including macrocephaly, frontal bossing, hypertelorism, skeletal defects, palmar pits and predisposition for BCC and medulloblastoma development [209]. Mutations have been identified in hedgehog components, mostly located to *PTCH1*. Also, in sporadic BCC and medulloblastoma, mutations in Hh-components, including *PTCH2*, *SUFU* and *SMO*, have been demonstrated [210]. As described above, hedgehog-signaling is dependent on intact cilia to appropriately process GliA and GliR.

One of the most frequently mutated genes in somatically acquired colorectal cancer is the *APC* tumor suppressor which, if mutated in the germline, causes familial adenomatous polyposis (FAP). One subtype of FAP is Gardner’s syndrome characterized by frequent extracolonic manifestations such as osteomas, skin cysts, desmoid tumors and retinal abnormalities [211]. This disease spectrum overlaps with ciliopathy disease characteristics and suggests a role for APC in cilia function. In addition, retrospective studies noted that most patients with FAP display extracolonic ciliopathy phenotypes of varying severity [211]. APC can bind the kinesin-2 member Kif3a and microtubule binding protein EB1, both essential for ciliation [212,213]. In addition, APC interacts with EB1 and KIF17 and stabilizes microtubule plus-ends [214].

In addition to the tumor suppressors in familial cancer development described above, a number of additional tumor suppressors and oncogenes, not directly associated with familial cancer syndromes but often somatically mutated in sporadic cancers, also can affect cilia biogenesis. Members of the Wnt- and Hh-signaling pathway that have somatically acquired oncogenic activity such as β-catenin, Ptc, Smo, Sufu and Gli lead to aberrant transcription of target genes, which is a constituent in many cancer types [68]. The NEK family is composed of serine-threonine kinases involved in cell cycle regulation and cancer formation. NEK1, NEK2 and NEK8/NPHP9 are related to ciliary biogenesis [215,216]. Mouse models affecting either NEK1 or NEK8 protein function show phenotypic overlap with ciliopathies [217,218]. Inactivation of *NEK1* was recently associated with the human ciliopathy autosomal-recessive short-rib polydactyly syndrome [219]. Mutations in *NEK1*, *NEK2* and *NEK8* have been identified in liver, ovarian, GI and lung cancers [215]. Furthermore, NEK8/NPHP9 was recently shown to directly bind the oncogenic transcriptional activator TAZ [157], as was also reported for transition zone component NPHP4 [106]. These findings are exciting since the Hippo pathway comprises a number of proteins that act as a tumor suppressor network in the control of organ size and growth control [105].

### Chromosomal instability and cilia

The cell makes use of an elaborate and elegant system of checkpoints to prevent and counteract cellular transformation and cancerous outgrowth. These include the mitotic or spindle checkpoint, as well as the DNA-damage checkpoints. Failure of these control systems leads to an accumulation of errors that eventually will result in chromosomal instability, a major driver of cancer progression. With the exception of certain terminally differentiated cell types, only one centrosome is present per quiescent cell. Upon duplication in S-phase, centrosomes migrate to opposing sides of the cell where they form spindle poles during mitosis. Although cells are still able to successfully segregate their chromosomes in the complete absence of centrosomes, centrosomal abnormalities can have severe effects on cells, ranging from defective spindle orientation, ineffective asymmetric cell division and cancer. Defects in centrosome separation or overreplication of centrosomes drive aneuploidy [116]. Similarly, when cytokinesis is perturbed, cells can inherit abnormal centrosome numbers. In addition, cells inheriting multiple centrosomes will form an equivalent number of cilia, which will distort signal interpretation [220]. Centrosomal abnormalities are a prominent feature of human tumors [220]. There are obviously many regulators of correct centrosome duplication and cytokinesis; here we will only discuss the crossroads between cilia biology and cancer development. Cilia defects in the kidney often lead to cyst formation, and these cysts can be considered benign neoplasms as they present hyperplasia; however, the incidence of tumor development in patients with cystic ciliopathies is low, and a second event is likely required for tumor growth initiation.

There is some emerging evidence that cilia defects can be linked to mitotic abnormalities. Murine endothelial cells derived from *Pkd2* and *Orpk/Ift88* knockout mice in addition to cell samples derived from human ADPKD patients had multipolar spindles and centrosomal abnormalities are observed that lead to polyploidy and chromosomal instability. Further analysis of these cells indicated that the chromosomal passenger complex protein survivin was down-regulated [221]. As discussed above, BUBR1 functions both in mitotic and ciliogenesis control [207], and interestingly, another member of the spindle assembly checkpoint, Mps1, has recently been shown to also affect ciliogenesis [222]. In cycling cells, depletion of either Mps1 or its direct interaction partner VDAC3 induces aberrant ciliogenesis, and upon serum starvation localization of Mps1 to the centrosome is sufficient to suppress ciliogenesis in RPE cells, suggesting it is a negative regulator of ciliation [222]. It would be interesting to study other components of the spindle checkpoint, such as the MAD proteins, for putative roles in ciliogenesis. Several other cell culture-based studies support a mechanism that could explain the induction of centrosomal abnormalities in cilia-related proteins; we previously reported that there is a large proteomic overlap between ciliogenesis and cytokinesis, including members of the chromosomal passenger complex [126], indicating that defects in these proteins can affect ciliogenesis as well as mitosis. As described in more detail above, oncogenic activation of HEF1 and downstream Aurora A induce premature cilia resorption and induce centrosomal over-replication [127]. Indeed, disruption of several cilia-related genes stimulates inappropriate cell cycle progression that can develop into replication stress. The IFT motor kinesin-2 does not exclusively function in cilia, but is also pivotal for normal mitosis; mutant KIF3B induces aneuploidy and induces multipolar spindle formation in NIH3T3 cells [223]. In addition, the KIF3 complex functions as a tumor suppressor in embryonic brain tumors, and *Kap3-/-* MEFs fail to transport cadherin complexes to the membrane and to inhibit downstream β-catenin activity [224].

The accumulation of DNA damage over time similarly increases the risk of cell transformation and cancer development. Cellular checkpoints normally prevent cells from proliferation under these conditions; this is regulated through the DNA damage response pathway, which is largely mediated by ATM and ATR [225]. The centrosomal proteins CEP152 and PCNT are required for ATR-dependent DNA damage response-signaling. Mutations in these genes lead to Seckel syndrome that is characterized by dwarfism, microcephaly and mental retardation, phenotypically overlapping somewhat with the ciliopathy disease spectrum [226]. Recently, we and others have shown that mutations in *SDCCAG8*, *ZNF423* and *CEP164* cause a NPHP-related ciliopathy and that these proteins localize to both the centrosome and DNA damage foci, and are required for proper DNA damage-induced response [226,227]. CEP164 is a well known regulator of cilia formation and is one of the earliest proteins that defines a matured centriole by the formation of distal appendages [228]. In addition, we have seen that Cep164 loss not only induces DNA damage response but also induces DNA damage in the form of lagging anaphases, probably resulting in mitotic catastrophe. Induced DNA damage by irradiation causes premature centriole splitting [229]. The resulting centriolar clusters sustain ciliogenesis, but upon depletion of centriolar linker proteins C-NAP1 or rootletin, ciliogenesis is perturbed [230]. It would be of interest to further study the defective cilia/DNA damage response crosstalk in light of cancer biology.

### Metastasis

Cell migration occurs generally in early development, wound-healing and chemotaxis. In aggressive cancers, transformed cells detach from the primary tumor and migrate throughout the body to form distant metastases. Cell migration is a multi-step process that partially depends on highly dynamic actin rearrangements, loss of focal adhesions, lamellipodia formation and changes of cell-matrix interaction [231]. In this light it is also of interest that Aurora A activator HEF1 regulates focal adhesions and is a prometastatic factor with elevated expression in melanoma and glioblastomas [127]. Cilia regulate normal cell migration in wound healing in response to the PDGF-AA chemokine, and fibroblasts defective in primary cilia fail to initiate chemotaxis [102]. It was observed that cilia are often oriented towards the direction of migration, suggesting that cilia provide context for directed cell migration [103]. Endothelial cells derived from the *Ift88-/-* oak ridge polycystic kidney mouse model showed reduced F-actin stress fibers, reduced focal adhesions and impaired cell migration due to down-regulated Hsp27 and focal adhesion kinase (FAK). Endothelial cells derived from Pkd1 (PC1) deficient mice did not show these defects, indicating that normal ciliation is involved in this process [232]. Finally, in an RNA interference screen in MCF-10A cells, NEK8 depletion was identified as a potent inducer of induced cell migration [233]. There are however many different signal transduction pathways that at various levels contribute to cellular migration, such as cell-matrix interactions, cell polarity and receptors dispersed across the plasma membrane rather than being enriched in the cilium. In the years to come, there will likely be an expansion of our understanding of the role of cilia in cell migration, and hence how, and if, defects in ciliary-signaling might affect cancer metastasis formation.

### Conclusions/perspectives

**Figure 5:**
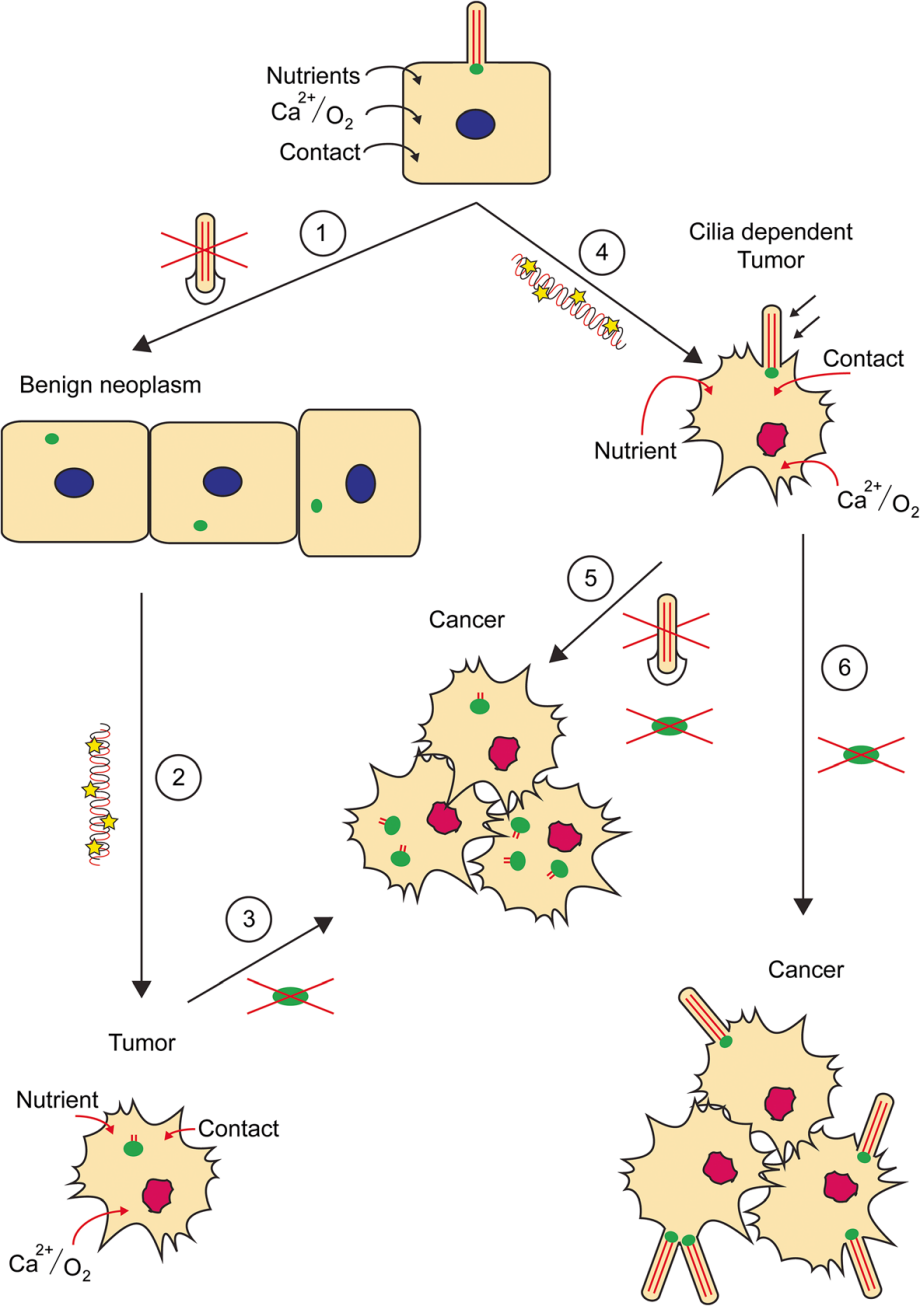
**Model of cilia and cancer. **We propose a number of pathways that can lead to cilia-dependent and cilia-independent tumor formation. The left pathway describes the oncogenic events that must follow after cilia have been lost **(1) **and a benign neoplasm has been formed; accumulation of genetic lesions **(2) **that desensitize cells to the microenvironment or CIN **(3) **to drive further cancer progression. Alternatively, the right pathway indicates a scenario where cilia are not involved in the initial transformation event **(4)**. Depending on the underlying oncogenic mechanism, cells will further develop into a cilia-independent cancer **(5)**, or will select for cilia retention in cilia-dependent cancer types **(6)**.

Because of the tremendous efforts made to dissect ciliary function, the level of data depth has now reached a point where we can appreciate the subtle reality that the cilium both taps into and cross-talks with many classical molecular signaling pathways, coordinating and fine-tuning cellular responses. Cancer development is a multi-step process that does not depend on one singular event, but is suggested to require the sequestration of at least six capabilities that allow survival and outgrowth. These include (1) desensitization to anti-proliferative signals and (2) replicative stress, (3) escape from apoptosis, (4) acquisition of sustained angiogenesis and (5) metastasis potential and (6) autonomous generation of proliferative signals. To achieve this, cancer cells must promote chromosomal instability followed by continuous trial-and-error before the required capabilities have been acquired [234]. Therefore the proclamation that tumorigenesis is a *bona fide* ciliopathy, or whether the cilium is a *bona fide* tumor suppressor, can be rejected, as neither of the two is correct. Depending on the oncogenic event, cells will either select for cilia retention or disposal and are thus upstream or downstream of cancer development. In Figure 5 we describe ways the cilium might function in tumorigenesis. Most cells normally establish a cilium and are able to interpret environmental sensing such as contact signals or metabolic factors including nutrient, oxygen and Ca^2+^ levels. Step 1: cilia are lost and will result in a benign neoplasm with limited over-proliferation and polarity defects. Step 2: environmental sensing is lost and cells transform. Step 3: cumulative DNA damage and replicative stress induce chromosome instability and lead to malignant cancer. Based on the clinical observation described above, the trend in most cancers seems to be in accordance with steps 1 to 3. Step 4: alternatively, cells transform but remain dependent on ciliary signaling. Steps 5 and 6: these tumors can acquire chromosomal instability and either dispose of (5), or retain (6), cilia to become cancerous. Cataloging various tumor subtypes and relating these to ciliation frequency scores might be an important next step in relation to future therapeutic approaches. Tumors that depend on cilia function, for example Hh-mediated tumors, might benefit from complementary treatment with Aurora A activators which promote cilia disassembly and therefore dampen Hh-signaling, or targeting downstream Hh transcription factors. Alternatively, tumors that select for cilia disposal might benefit from treatment with cilia-stabilizing molecules such as low-dose taxol and HDAC-inhibitors. Low-dose paclitaxel stabilizes microtubules and cilia and ameliorates renal fibrosis in rats [235]. For extensive reviews on putative therapeutic approaches we refer to excellent surveys by Scales and de Sauvage [168] and Hassounah, Bunch and McDermott [151]. Thus, although the cilium does not single-handedly drive or prohibit tumorigenesis by default, targeting its function could be a method to switch cancer off.

## Abbreviations

ACKD: Acquired cystic kidney disease; BB: Basal body; CNS: Central nervous system; GAP: Guanine activating proteins; GEF: Guanine exchange factors; Hh: Hedgehog-signaling; IFT: Intraflagellar transport; PCP: Planar cell polarity; TZ: Transition zone.

## Competing interests

Both authors declare that they have no competing interests.

## Authors’ contributions

SB and RG wrote the paper. Both authors read and approved the final manuscript.
